# Combined Cytotoxicity of the Phycotoxin Okadaic Acid and Mycotoxins on Intestinal and Neuroblastoma Human Cell Models

**DOI:** 10.3390/toxins10120526

**Published:** 2018-12-08

**Authors:** Aiko Hayashi, Juan José Dorantes-Aranda, John P. Bowman, Gustaaf Hallegraeff

**Affiliations:** 1 Institute for Marine and Antarctic Studies, University of Tasmania, 7004 Hobart, Australia; juan.dorantesaranda@utas.edu.au (J.J.D.-A.); Hallegraeff@utas.edu.au (G.H.); 2 Tasmanian Institute of Agriculture, University of Tasmania, 7005 Hobart, Australia; john.bowman@utas.edu.au

**Keywords:** okadaic acid, sydowinin A, sydowinol, alamethicin, patulin, gliotoxin, combination index, synergy

## Abstract

Mycotoxins are emerging toxins in the marine environment, which can co-occur with algal toxins to exert synergistic or antagonistic effects for human seafood consumption. The current study assesses the cytotoxicity of the algal toxin okadaic acid, shellfish, and dust storm-associated mycotoxins alone or in combination on human intestinal (HT-29) and neuroblastoma (SH-SY5Y) cell lines. Based on calculated IC_50_ (inhibitory concentration 50%) values, mycotoxins and the algal toxin on their own exhibited increased cytotoxicity in the order of sydowinin A < sydowinin B << patulin < alamethicin < sydowinol << gliotoxin ≈ okadaic acid against the HT-29 cell line, and sydowinin B < sydowinin A << alamethicin ≈ sydowinol < patulin, << gliotoxin < okadaic acid against the SH-SY5Y cell line. Combinations of okadaic acid–sydowinin A, –alamethicin, –patulin, and –gliotoxin exhibited antagonistic effects at low-moderate cytotoxicity, but became synergistic at high cytotoxicity, while okadaic acid–sydowinol displayed an antagonistic relationship against HT-29 cells. Furthermore, only okadaic acid–sydowinin A showed synergism, while okadaic acid–sydowinol, –alamethicin, –patulin, and –gliotoxin combinations demonstrated antagonism against SH-SY5Y. While diarrhetic shellfish poisoning (DSP) from okadaic acid and analogues in many parts of the world is considered to be a comparatively minor seafood toxin syndrome, our human cell model studies suggest that synergisms with certain mycotoxins may aggravate human health impacts, depending on the concentrations. These findings highlight the issues of the shortcomings of current regulatory approaches, which do not regulate for mycotoxins in shellfish and treat seafood toxins as if they occur as single toxins.

## 1. Introduction

The importance of fungi in the marine environment has been increasingly recognised in recent years. They are capable of infecting a wide range of marine animals, including sea turtles [1] and sea fan corals [2], and threatening human health through mycotoxin accumulation in seafood [3]. The majority of infectious fungi in the marine environment are considered to be of terrestrial origin [4], but atmospheric dust deposition and terrestrial runoff can facilitate the growth of fungi already residing in the marine environment and/or introduce them from terrestrial into marine environments. For example, an *Aspergillus sydowii* “bloom” (150,000 spores/m^2^) along the east coast of Australia was observed after an extensive dust storm in 2009 [5]. Similarly, increased dust deposition and nutrient input from terrestrial runoff is thought to have contributed to an outbreak of the fungal disease sea fan coral aspergillosis in the Caribbean [6].

Fungal contaminants in seafood can also pose a significant human health risk. Several studies have shown that toxigenic fungal species can reside within the shellfish itself, seawater, and sediments from aquaculture farming areas. *Penicillium*, *Aspergillus*, *Trichoderma*, and *Cladosporium* have been isolated from such samples in France [7], Canada [8], Algeria [9], Russia [10], Brazil [11], Italy [12], and Tunisia [13]. These genera of fungi are capable of producing toxic metabolites (mycotoxins), including aflatoxins (AF), zearalenone (ZEA), deoxynivalenol (DON), fumonisins (FB), and ochratoxins (OTA) [14]. These compounds exhibit a wide range of biological activities, including hepatocarcinogenic, genotoxic, carcinogenic, oestrogenic, nephrotoxic, and nephrocarcinogenic effects [15]. Evidence exists that some shellfish-associated fungal isolates were capable of producing highly toxic mycotoxins, such as gliotoxin by *Aspergillus fumigatus* [3], patulin by *Penicillium* sp. [16], peptaibol by *Trichoderma* sp. [17], and griseofulvin by *P. waksmanii* [18]. These mycotoxins have been demonstrated to bio-accumulate in shellfish under both laboratory and natural conditions. A filtrate of marine-derived *T. koningii*, gliotoxin accumulated in shellfish, and peptaibols were detected in shellfish and sediments from aquaculture environments [3,17,19]. C17-sphinganine analogue mycotoxin (C17-SAMT) was claimed to be solely responsible for high shellfish toxicity in Tunisia in 2006 [13]. Mycotoxins are now widely viewed as new emerging toxins in shellfish.

Mycotoxins on their own can pose a significant health risk for humans through shellfish consumption, but an even greater concern arises from their possible synergistic effects with co-occurring algal toxins. However, mycotoxins in shellfish are currently not monitored and information on the combined effects of algal toxins and mycotoxins is sparse. So far, an in vivo Diptera larval bioassay by Ruiz et al. has been the only study to assess the combined effects of the algal toxin domoic acid and mycotoxin. Their study revealed increased toxicity by up to 34.5 times (the synergism factor) when domoic acid and longibranchi-A-I were injected together into Diptera larvae [20]. The proposed mechanism of this synergism was enhanced by an increase in Ca^2+^ influx into the cells by both domoic acid and novel peptaibol longibranchi-A-I [20].

**Figure 1:**
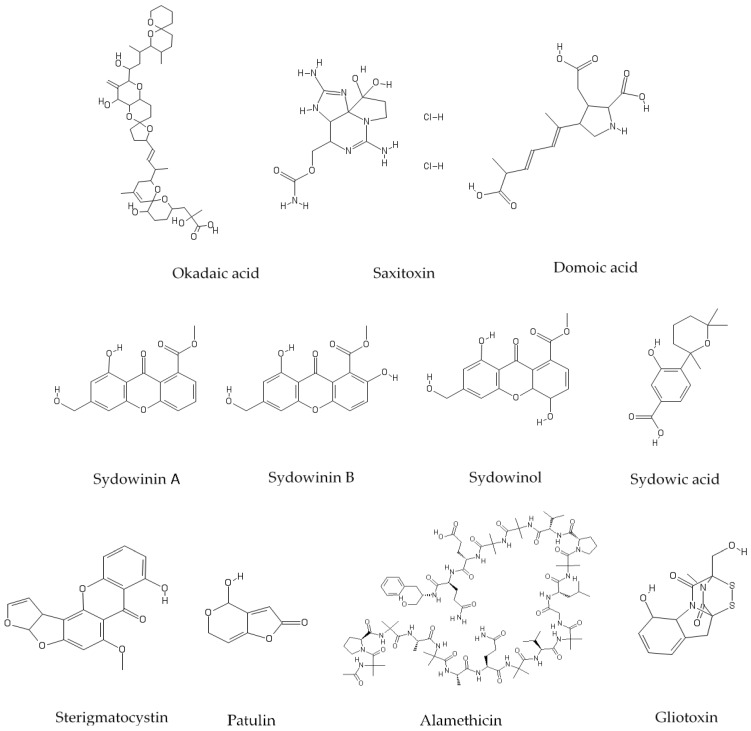
Chemical structures of typical algal toxins (okadaic acid, saxitoxin, domoic acid), dust storm-related mycotoxins (major *A. sydowii* metabolites and sterigmatocystin), and shellfish-related mycotoxins (patulin, alamethicin, gliotoxin).

The management of seafood safety is important for public health, market access, and public confidence. For example, a single incident of failure of detecting unacceptable levels of paralytic shellfish toxins (PST) in exported mussels resulted in AUD$24 million dollar economic loss to the Tasmanian seafood industry [21]. Current approaches to seafood safety management do not regulate for mycotoxins, and take no account of combined effects of co-occurring seafood toxins and treat them as if they were to occur as individual compounds [22,23]. Therefore, the aim of this study was to identify the toxic interactions of major algal toxins (e.g., saxitoxin, domoic acid and okadaic acid) and shellfish-associated (e.g., gliotoxins, patulin and peptaibol) and dust-originated (*A. sydowii* metabolites and sterigmatocystin [24]) mycotoxins (Figure 1) using human intestinal HT-29 and neuroblastoma SH-SY5Y cell line models. HT-29 and SH-SY5Y were chosen for assessing gastrointestinal and neurological effects, respectively, from saxitoxin [25], domoic acid [26] and okadaic acid [27]. Toxin interactions such as synergisms, antagonism, and additive were quantitatively evaluated with the combination index (CI) method [28].

## 2. Results

### 2.1. Individual Cytotoxicity of Algal Toxin and Mycotoxin

The cytotoxicity of individual mycotoxins and phycotoxins on the human intestinal cell line HT-29 and human neuroblastoma cell line SH-SY5Y was evaluated using resazurine cell viability reagent. The tested mycotoxins, except sydowic acid, exhibited a dose-dependent effect with a range of inhibitory concentration 50% (IC_50_) from 65 nM to 124 µM for HT-29, and from 45 nM to 144 µM for SH-SY5Y (Table 1 and Supplementary Data in Figures S1 and S2). The tested concentration ranges of sydowic acid (HT-29: 0.028–283.75 µM, SH-SY5Y: 0.567–567.49 µM) showed no significant effect on viability for both HT-29 and SH-SY5Y (HT-29: *F*(8,27) = 0.095, *p* = 0.999, SH-SY5Y: *F*(4,15) = 1.516, *p* = 0.248). Sterigmatocystin reduced the viability of both HT-29 and SH-SY5Y in a dose-dependent manner with an incomplete sigmoid curve. The highest applicable concentration of 62 and 123 µM sterigmatocystin lowered the viability of HT-29 to 60%, and that of SH-SY5Y to 43%, respectively. Therefore, the IC_50_ of sterigmatocystin was not calculated. Okadaic acid displayed a dose-dependent effect on HT-29, with IC_50_ of 65 nM, and SH-SY5Y viability, with IC_50_ of 27 nM, whereas the other tested algal toxin, saxitoxin, and domoic acid had either no effect or minor effects on the viability of HT-29 and SH-SY5Y (maximum tested concentrations were 16.6–1.33 µM) (Figures S1 and S2). For the overall cytotoxicity ranking, based on the calculated IC_50_ values, the tested mycotoxin and algal toxin were found to be in the increasing order of sydowinin A < sydowinin B << patulin < alamethicin < sydowinol << gliotoxin ≈ okadaic acid in HT-29, and sydowinin B < sydowinin A << alamethicin ≈ sydowinol < patulin, <<gliotoxin < okadaic acid in SH-SY5Y.

### 2.2. Combined Cytotoxicity of Mycotoxins and Algal Toxin

Since okadaic acid was the only algal toxin which exhibited cytotoxicity on both HT-29 and SH-SY5Y cells, the effects of combined okadaic acid and mycotoxin sydowinin A, sydowinol, patulin, alamethicin, and gliotoxin on cell viability of HT-29 and SH-SY5Y were examined. Sydowinin B, sydowic acid, and sterigmatocystin were eliminated from the combined cytotoxicity assay because of their low cytotoxicity and limited solubility. Furthermore, the combination ratios were chosen to have an equipotent toxicity of each toxin (e.g., (IC_50_)_1_/(IC_50_)_2_ ratio) (Table 2), as there were no data available on the concentration of mycotoxins in shellfish, and this was recommended by Chou for an early stage study [29]. The combination index (CI) values were calculated from a fraction of cell viability affected (*fa*) values of 0.05 (corresponding to IC_05_) to 0.97 (corresponding to IC_97_), and the dose reduction index (DRI) was calculated when synergistic interactions were detected. All the binary mixtures of toxins showed a dose-dependent effect on HT-29 and SH-SY5Y cells (Figures S3 and S4).

### 2.3. Okadaic Acid and Mycotoxins on Human Intestinal HT-29 Cells

**Figure 2:**
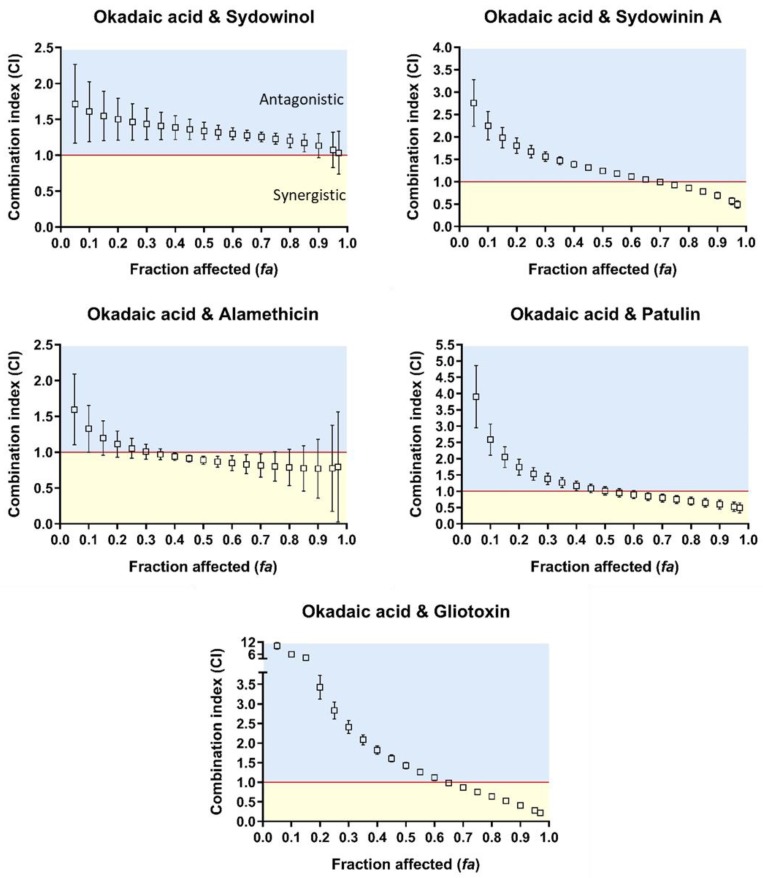
Combination index (CI)–fraction affected (*fa*, indicating fraction of cell viability affected. *fa* = 0.05–0.97 corresponds to 5–97% toxicity) curves for binary mixtures of okadaic acid and sydowinol, sydowinin A, alamethicin, patulin, and gliotoxin against human intestinal HT-29 cells. CI < 1, CI = 1, and CI > 1 indicate synergistic (orange rectangle), additive (red line), and antagonistic (blue rectangle) effects of binary mixtures, respectively. The error bar indicates 95% confidence intervals calculated using sequential deletion analysis (SDA).

Okadaic acid–sydowinin A, –alamethicin, –patulin, and –gliotoxin binary mixtures displayed variations of the interaction types on human intestinal HT-29 cells dependent upon the effect levels (Figure 2). At low to moderate effect levels (*fa* < 0.65), these combinations exhibited antagonistic to additive effects, while they presented synergistic relationships at higher effect levels (*fa* > 0.65). In contrast to these okadaic acid–mycotoxin mixtures, okadaic acid–sydowinol mixtures displayed antagonistic effects at *fa* > 0.95 and a nearly additive interaction at *fa* < 0.95 (Figure 2). The DRI values for okadaic acid and mycotoxins varied from 1.8 to 12.5 and 1.8 to 12.2, respectively (Table 3). The greatest synergistic effect at *fa* = 0.9 was noted for the binary mixture of okadaic acid and gliotoxin, with a CI value of 0.41. For this combination, at the effect level of 0.9, the okadaic acid and gliotoxin mixture was 12.4 times more potent than okadaic acid alone, and 3 times more effective than gliotoxin alone.

### 2.4. Okadaic Acid and Mycotoxins on Human Neuroblastoma SH-SY5Y Cells

**Figure 3:**
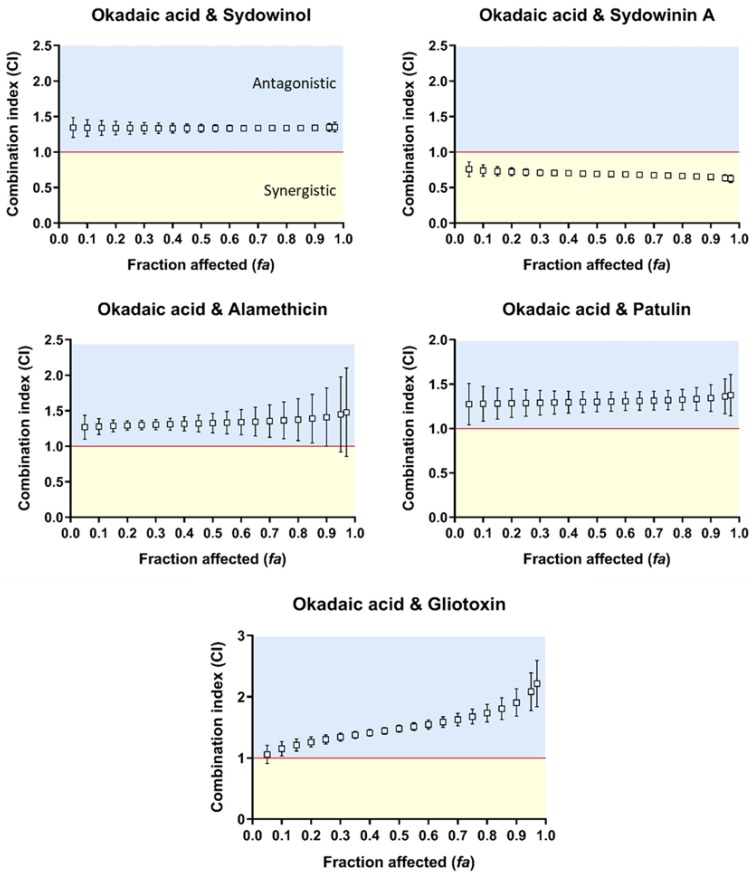
Combination index (CI)–fraction affected (*fa*, indicating fraction of cell viability affected. *fa* = 0.05–0.97 corresponds to 5–97% toxicity) curves for binary mixtures of okadaic acid and sydowinol, sydowinin A, alamethicin, patulin, and gliotoxin against human neuroblastoma SH-SY5Y cells. CI < 1, CI = 1, and CI > 1 indicate synergistic (orange rectangle), additive (red line), and antagonistic (blue rectangle) effects of binary mixtures, respectively. The error bar indicates 95% confidence intervals calculated using sequential deletion analysis (SDA).

Okadaic acid–sydowinol, –alamethicin, –patulin, and –gliotoxin mixtures on human neuroblastoma SH-SY5Y cells showed an antagonistic interaction type at all effect levels, except that at *fa* = 0.05; gliotoxin and okadaic acid exhibited an additive interaction type (Figure 3). The calculated CI values for these combinations varied from 1.15 to 2.21 (Figure 3). By contrast, okadaic acid–sydowinin A mixtures exhibited synergisms at all effect levels, with a CI of 0.65 at *fa* = 0.9. For this combination, at the effect level of 0.9, the okadaic acid and sydowinin A mixture was 3.3 times more effective than okadaic acid alone and 2.9 times more effective than sydowinin A alone (Table 3).

## 3. Discussion

We demonstrated in this study that binary mixtures of the phycotoxin okadaic acid, and dust- and shellfish-associated mycotoxins exhibited cell line- and concentration-dependent antagonistic or synergistic interactions. Combinations of okadaic acid–sydowinin A, –alamethicin, –patulin, and –gliotoxin exhibited synergisms at higher effect levels and antagonisms at lower effect levels on HT-29. Interestingly, only okadaic acid–sydowinin A displayed synergism, whereas antagonism was noted for other combinations on SH-SY5Y at all effect levels. DRI values indicated that toxin doses can be theoretically reduced by up to 1.8 to 12-fold for the combination to have the same effect as that induced by each toxin on its own. These findings suggested that ingestion of a regulatory safe level of the algal toxin okadaic acid (0.16 mg OA eq./kg) could result in a health impact due to synergism with mycotoxin.

### 3.1. Synergisms between Okadaic Acid and Mycotoxins

We speculate that synergistic effects of okadaic acid and the tested mycotoxins on HT-29 could be the result of the impairment of cell structure. Okadaic acid is the main lipophilic marine biotoxin produced by *Dinophysis* and *Prorocentrum* dinoflagellates and responsible for diarrhetic shellfish poisoning (DSP) in humans [30]. Okadaic acid is an inhibitor of serine/threonine protein phosphatases (PP), which affect various important cellular metabolic processes, leading to cytoskeleton and intestinal mucosa deterioration, digestive dysfunction, lipid metabolism disorders, oxidative stress, and cellular apoptosis [31]. These series of events contribute to the gut barrier impairment and intestinal cell degeneration, which results in human diarrheic symptoms [31]. The mycotoxin alamethicin, also known as peptaibol, forms pores in membranes, thereby increasing membrane permeability [32]. Similarly, gliotoxin specifically binds to cytoplasmic membrane thiol groups, causing an increase in membrane permeability by affecting membrane protein orientation [33]. Patulin also induces the depletion of nonprotein sulfhydryl groups and increases potassium efflux, which results in the loss of structural integrity of the plasma membrane [34]. While mycotoxins have different mechanisms of action, they all lead to a disruption of ion homeostasis and structural damage which in turn potentially compounds downstream effects caused by okadaic acid in particular cytoskeleton deterioration, oxidative stress, and apoptosis. Furthermore, the observed shifts from antagonism to additive/synergism with increasing concentrations in the current study have also been reported in the similar study, where the interaction types of lipophilic phycotoxins (e.g., okadaic acid, pectenetoxin-2, yessotoxin, spirolide-1) were examined [35].

Okadaic acid and sydowinin A exhibited synergistic effects on both the HT-29 and SH-SY5Y cell lines. Currently, we lack knowledge of the details of the mode of action of the major *Aspergillus sydowii* metabolites sydowinin A and sydowinol. Sydowinin A has been reported to have more potent immunosuppressive effects on the Con A-induced and lipopolysaccharide-induced proliferations of mouse splenic lymphocytes compared to other *A. sydowii* metabolites [36]. The current study and other studies supported evidence of that the okadaic acid-induced PP inhibition also induces various neurotoxic effects [37,38]. However, no major human neurotoxic symptoms from ingesting okadaic acid-contaminated seafood have been reported so far, probably due to the levels of okadaic acid accumulating more slowly in the brain compared to the stomach and gastrointestinal tract tissues [39]. Synergistic relationships between okadaic acid and sydowinin A may have a basis in that the immunosuppressive characteristics of sydowinin A could sensitise cells to okadaic acid, but this requires investigation. The observed synergistic relationships with sydowinin A imply that even a low level of okadaic acid may cause significant neurotoxic effects in humans.

### 3.2. Antagonisms between Okadaic Acid and Mycotoxin on SH-SY5Y

The combination of okadaic acid and the tested mycotoxins exhibited antagonistic relationships against SH-SY5Y neuroblastoma cells, whereas interactions were synergistic against HT-29 intestinal cell lines. Antagonistic interactions were also noted for HT-29 at the low effect level. These observed antagonisms could be explained by multidrug resistance (MDR). MDR is regulated by P-glycoprotein (P-gp), which functions as an efflux transport pump, removing toxins from the plasma membrane, hence reducing cytotoxicity [40]. Okadaic acid efflux occurred in okadaic acid-resistant Chinese hamster ovary cells with increased levels of P-gp [41]. Therefore, the observed antagonisms in SH-SY5Y cells could be related to less mycotoxin binding to the target site, while okadaic acid is actively removed from the plasma membrane. This could lead to lower toxicity than estimated for the combined effect. This is supported by the fact that undifferentiated SH-SY5Y cells expressed some degree of P-gp expression, while HT-29 showed no detectable P-gp [42,43]. Furthermore, in the present study, mycotoxins were more abundant than okadaic acid in the binary mixtures, which could make mycotoxins more readily bind to the target site. Similarly, Alassane-Kpembi et al. (2015) suggested that the MDR drug efflux mechanism might explain the observed antagonism between deoxynivalenol (DON)–3-Acetyldeoxynivalenol (3-ADON) and DON–Fusarenon-X (FX) combinations [44]. However, the suggested mechanisms of antagonisms remain speculative and require further study.

## 4. Conclusions

The present study demonstrated that binary mixtures of okadaic acid and shellfish- and dust-associated mycotoxins displayed cell line- and concentration-dependent interactions. The general interaction patterns observed in this study were a shift from antagonism to synergism with increasing concentrations on HT-29 cells, and antagonism or synergism at all concentrations on SH-SY5Y cells. The synergistic effects observed in the current study are of practical significance. While diarrhetic shellfish poisoning from okadaic acid and analogues is widely considered to be a comparatively minor seafood toxin syndrome (e.g., no human fatalities have ever occurred), our human cell model studies provided preliminary insights that synergisms with mycotoxins can be expected to more seriously aggravate human health impacts.

This also suggests the need for implementing more studies of seafood where there is risk of the co-occurrence of mycotoxins and algal toxins. Our results clearly demonstrate that the toxin interaction type depends on the effect level and cell type. This points to difficulties of predicting toxin interactions from the known mechanisms of actions of individual toxins without actual experimental data [29]. Mycotoxins are emerging toxins in seafood, and their occurrence may increase due to increased terrestrial runoff, dust storms, and the use of mycotoxin contaminated aquaculture feeds [23]. The current study did not explore the precise cellular mechanisms behind the mycotoxin and algal toxin interaction, and suggested mechanisms therefore remain speculative, and deserve further study. Future work should prioritise determining the interaction types of commonly occurring algal toxins (e.g., saxitoxin and domoic acid), and other mycotoxins (e.g., DON, AF, ZEA, FB, and OTA) [45]. Multiple mixtures (e.g., more than two toxins) should also be considered. Our results highlight the possible risks of toxin co-occurrence in seafood, a scenario which is not considered in current shellfish safety regulations.

## 5. Materials and Methods

### 5.1. Cell Line Cultures

Human neuroblastoma SH-SY5Y was kindly provided by Ms Yilan Zhen and Dr. Kaylene Young (Menzies Institute for Medical Research, University of Tasmania, Australia). Human colorectal adenocarcinoma cells HT-29 were kindly provided by Dr. Anthony Baker (Tasmanian Institute of Agriculture, University of Tasmania and School of Land and Food, Australia). Both cell lines were routinely maintained in Dulbecco’s Modified Eagle’s Medium (DMEM, D0819, Sigma-Aldrich, Sydney, Australia) supplemented with 10% foetal bovine serum (FBS, Bovogen Biologicals, Melbourne, Australia), and 100 U/mL penicillin and 100 mg/mL streptomycin solution in a humidified incubator (5% CO_2_, 37 °C). SH-SY5Y cells were routinely subcultured at a ratio of 1:30–1:50, and medium changeover occurred approximately every 5 d. HT-29 cells were routinely subcultured at a ratio of 1:3–1:8, and medium changeover occurred approximately every 4 d.

### 5.2. Mycotoxin and Phycotoxin Toxins

Four typical *Aspergillus sydowii* metabolite standards, sydowinin A, sydowinin B, sydowinol, and sydowic acid were kindly provided by Professor Hiromitsu Nakajima, Tottori University, Japan. These compounds were isolated from *A. sydowii* IFO 4284 and IFO 7531 strains. Full descriptions of UV, IR, and NMR spectra, chemical structures, and molecular weights of these metabolites were previously provided by Hamasaki et al. (1975a,b) [46,47]. The crystallised *A. sydowii* metabolites were weighted on a microbalance and dissolved in small volumes of acetone (>0.5 mL). Among the other fungal toxins tested, gliotoxin (G9893, Sigma-Aldrich) was dissolved in ethanol, alamethicin (A4665, Sigma-Aldrich) was dissolved in DMSO, and sterigmatocystin (S3255, Sigma-Aldrich) and patulin (P1639, Sigma-Aldrich) were dissolved in acetonitrile. Phycotoxin standards, saxitoxin (CRM-STX-f), domoic acid (CRM-DA-g), and okadaic acid (CRM-OA-d) were purchased from the National Research Council Canada. Concentrations used are expressed as µM.

### 5.3. Cytotoxicity Bioassays

When cells reached >70% confluency, they were detached using a trypsin–EDTA solution. Detached cells were centrifuged 300 g for 5 min and resuspended. Cells were seeded to a 96-well plate at 1.0 × 10^4^ cells/well for HT-29 and 3.0 × 10^4^ cells/well for SH-SY5Y and allowed to attach for 24 h prior to toxin exposure. Each well contained 100 µL of cells suspension, and 0.5–3% (*v*/*v*) of algal toxin and mycotoxins stocks were added to the basal DMEM, which contained no supplemented FBS nor antibiotics. Concentration ranges of tested individual toxicity of algal toxins and mycotoxins were 1.33 × 10^−9^–123.3 µM for SH-SY5Y, and 3.12 × 10^−8^–235.6 µM for HT-29. For the combined cytotoxicity bioassay, the ranges were 0.019–214.9 µM for HT-29 and 0.016–169.6 µM for SH-SY5Y. Cells were rinsed once with DPBS (Dulbecco’s phosphate-buffered saline, 0.9 mM CaCl_2_; 0.50 mM MgCl_2_·6H_2_O; 2.7 mM KCl; 1.5 mM KH_2_PO_4_; 137.9 mM NaCl; 8.1 mM Na_2_HPO_4_·7H_2_O). Toxin-containing DMEM was added to each well and incubated further for 24 h. Controls received only solvents, and the solvent concentration used in the assay was preliminary tested to have no significant effect on the cell viability compared to those received basal DMEM without solvents (data not shown). After toxin exposure, the cells were washed once again with DPBS and 100 µL of the same basal media (without phenol red) containing 5% resazurin solution [48] were added to each well. Following additional 2 h incubation in the dark, the plate was read with a BMG FLUOstar OMEGA plate reader using excitation of 540 nm and emission of 590 nm. Cell viability was expressed as the percentage of fluorescence reading compared to the control (% of control). Four replicates were prepared for each treatment.

### 5.4. Statistical Analysis of Cytotoxicity of Individual Mycotoxin and Algal Toxin

Data analysis was conducted with the decision tree proposed by Sérandour et al. [49], except that in this experiment, the controls were preliminary tested to have no effect on cell viability and no further calculation was conducted when there was no bottom asymptote. Briefly, the dose response curves were fitted with the four-parameter logistic model (4PL), and 95% asymptotic confidence intervals were calculated using GraphPad Prism 7. The half-maximal inhibitory concentration (IC_50_) indicating the concentration that caused a half-maximal viability was calculated for each toxin. IC_50_ was accepted if the fitting dose–response curve had *R*^2^ > 0.85 and standard of error of log IC_50_ was <40%. One-way analysis of variance (ANOVA) was used to evaluate statistical differences between control and treatments. Tukey’s honestly significant different (HSD) post hoc tests were performed when the main effect was significant. Appropriate data transformation was determined using Box–Cox transformation. ANOVA and follow-up statistical analyses were performed with the statistical software R (R Development Core Team, version 3.4.3) [50]. A significance level of 0.05 was applied in this study.

### 5.5. Median Effect and Combination Index Analysis of Mycotoxin and Algal Toxin Mixture

The cytotoxicity of mycotoxin and algal toxin mixture was analysed based on the Chou–Talalay method [28]. The combination of mycotoxin and algal toxin were at an equipotency ratio (e.g., (IC_50_)_1_/(IC_50_)_2_ ratio) based on the calculated IC_50_ values using the graphpad prism 4PL model; therefore, each toxin roughly affects the cell viability equally [29]. The dose–responses for individual toxins and their mixture were modelled using the median effect equation of the mass action law:

(1) $$\frac{fa}{fu}={\left(\frac{D}{D_{m}}\right)}^{m}$$

where *D* is the dose of the toxin, *D_m_* is the median effect dose (e.g., IC_50_), *fa* is the fraction affected by dose (*D*) (e.g., fractions of cell viability affected), *fu* represents the fraction unaffected, and *m* indicates the shape of the slope (*m* = 1, > 1, and < 1 indicate hyperbolic, sigmoidal, and flat sigmoidal curves, respectively). Toxin interactions were only analysed when the linear correlation coefficient (*r*) of the median effect plot was greater than 0.92.

The mycotoxin and algal toxins interaction was analysed by the combination index (CI) method derived from the median effect equation of the mass action law. The combination index was calculated using the following equation below [29]:

(2) (CI)xn=∑j=1n(D)j(Dx)j

where (CI)xn is the combination index for n mycotoxins and algal toxins that inhibits x percent of a system (e.g., viability), ${\left(D\right)}_{j}$ are the doses that mixture of n phyco- and mycotoxins that inhibits x percent of a system, and ${\left(D_{x}\right)}_{j}$ are the doses that each phyco- and mycotoxin itself inhibits x percent of a system. CI < 1, = 1, and > 1 indicate synergism, additive effect, or antagonism, respectively. CI values were calculated over a range of *fa* = 0.05 to 0.97 (5–97% toxicity). A confidence interval of 95% (95% CI) for CI was calculated based on sequential deletion analysis (SDA). The dose reduction index (DRI) values were determined for the combination that exhibited a synergistic relationship at IC_25_, IC_50_, IC_75_ and IC_90_. DRI indicates the magnitude of how the dose of each drug in a mixture can be reduced at the given effect level compared to the doses of each drug alone. The dose–response analyses of toxin mixtures, CI, and DRI were performed with Compusyn software (ComboSyn Inc., Paramus, NJ, USA).

## Figures and Tables

**Table 1:** Summary of cytotoxicity of typical *A. sydowii* metabolites, dust storm/shellfish-associated mycotoxins, okadaic acid algal toxins on HT-29 and SH-SY5Y cells after 24 h exposure. Inhibitory concentration 50% (IC_50_) values and 95% confidence interval (CI) were calculated from four replicates using the four-parameter logistic model (4PL) model.

Toxin	HT-29	95% CI	SH-SY5Y	95% CI
	IC_50_ (µM)		IC_50_ (µM)	
*Typical A. sydowii metabolites*				
Sydowinin A	124.30	113.60–136.00	117.80	105.60–131.40
Sydowinin B	93.06	82.20–105.40	143.8	116.00–178.20
Sydowinol	2.50	2.21–2.82	5.14	5.06–5.23
Sydowic acid	NE (283.75) ^1^	-	NE (283.75) ^1^	-
*Dust storm/shellfish mycotoxins*				
Sterigmatocystin	>61.67 ^2^	-	~123.35 ^2^	-
Patulin	17.46	10.79–28.28	2.23	2.15–2.32
Alamethicin	4.92	4.57–5.29	5.43	5.29–5.67
Gliotoxin	0.062	0.052–0.075	0.045	0.039–0.053
*Algal toxins*				
Okadaic acid	0.065	0.056–0.075	0.027	0.026–0.029

^1^ NE indicates toxins had no significant effect within the tested concentration range. Numbers in brackets indicate the maximum applicable concentration tested. ^2^ The maximum applicable concentration of 61.67 µM and 123.35 µM sterigmatocystin lowered the viability to 60% on HT-29 and 43% on SH-SY5Y, respectively.

**Table 2:** Molar combination ratio of okadaic acid and mycotoxin mixtures used in the assay.

Toxin Mixture	Molar Combination Ratio	
	HT-29	SH-SY5Y
Okadaic acid:Sydowinin A	1:1925.0	1:14366.2
Okadaic acid:Sydowinol	1:38.7	1:190.7
Okadaic acid:Alamethicin	1:76.6	1:201.1
Okadaic acid:Patulin	1:270.4	1:82.6
Okadaic acid:Gliotoxin	1:1.04	1:1.68

**Table 3:** Combination index (CI) and dose reduction index (DRI) values for okadaic acid and mycotoxin combinations in HT-29 and SH-SY5Y cells at various effect levels (IC_25_, IC_50_, IC_75_ and IC_90_). DRI values were only calculated when synergistic effects were detected. DRI implies fold of dose reduction for a given effect in a combination of toxins compared with the dose of each toxin alone.

Toxin Mixture	CI at				DRI at			
	IC_25_	IC_50_	IC_75_	IC_90_	IC_25_	IC_50_	IC_75_	IC_90_
*HT-29*								
Okadaic acid	1.67	1.24	0.93	0.69	-	-	2.72	3.61
Sydowinin A					-	-	1.80	2.43
Okadaic acid	1.47	1.34	1.23	1.13	-	-	-	-
Sydowinol					-	-	-	-
Okadaic acid	1.06	0.88	0.78	0.72	-	2.41	2.18	1.98
Alamethicin					-	2.14	3.14	4.61
Okadaic acid	1.53	1.01	0.75	0.53	-	-	1.76	1.95
Patulin					-	-	5.63	12.23
Okadaic acid	2.84	1.42	0.75	0.41	-	-	4.80	12.45
Gliotoxin					-	-	1.85	3.02
*SH-SY5Y*								
Okadaic acid	0.72	0.69	0.67	0.65	2.98	3.09	3.20	3.32
Sydowinin A					2.65	2.72	2.79	2.86
Okadaic acid	1.34	1.33	1.34	1.34	-	-	-	-
Sydowinol					-	-	-	-
Okadaic acid	1.30	1.33	1.36	1.41	-	-	-	-
Alamethicin					-	-	-	-
Okadaic acid	1.29	1.30	1.32	1.34	-	-	-	-
Patulin					-	-	-	-
Okadaic acid	1.30	1.48	1.68	1.91	-	-	-	-
Gliotoxin					-	-	-	-

## Supplementary Materials

The following are available online at http://www.mdpi.com/2072-6651/10/12/526/s1, Figure S1: Dose–response curves of (a) major *A. sydowii* metabolites, (b) dust storm/shellfish mycotoxins, and (c) algal toxins on human intestinal HT-29 cells. Data are mean ± SD of four replicates. Figure S2: Dose–response curves of (a) major *A. sydowii* metabolites, (b) dust storm/shellfish mycotoxins, and (c) algal toxins on human neuroblastoma SH-SY5Y cells. Data are mean ± SD of four replicates. Figure S3: Okadaic acid (OA), sydowinol (SYD), sydowinin A (SYDA), alamethicin (ALA), patulin (PAT), and gliotoxin (GLI) and their binary mixture dose–responses for cytotoxicity against the human intestinal HT-29 cell line. Concentrations in combinations were expressed as the sum of the concentrations of two toxins. Data are mean ± SD of four replicates. Figure S4: Okadaic acid (OA), sydowinol (SYD), sydowinin A (SYDA), alamethicin (ALA), patulin (PAT), and gliotoxin (GLI) and their binary mixture dose–responses for cytotoxicity against the human neuroblastoma SH-SY5Y cell line. Concentrations in combinations were expressed as the sum of the concentrations of two toxins. Data are mean ± SD of four replicates.
