# Supplementary material for: Combined Cytotoxicity of the Phycotoxin Okadaic Acid and Mycotoxins on Intestinal and Neuroblastoma Human Cell Models
Source: Toxins (Basel). 2018 Dec 8;10(12):526. doi: 10.3390/toxins10120526 (PMC6315785; doi:10.3390/toxins10120526)
Supplement: Supplementary file 1 [file toxins-10-00526-s001.pdf]

toxins

Supplementary Materials: Combined Cytotoxicity of the Phycotoxin Okadaic Acid and Mycotoxins on Intestinal and Neuroblastoma Human Cell Models

Aiko Hayashi, Juan José Dorantes-Aranda, John P. Bowman and Gustaaf Hallegraeff

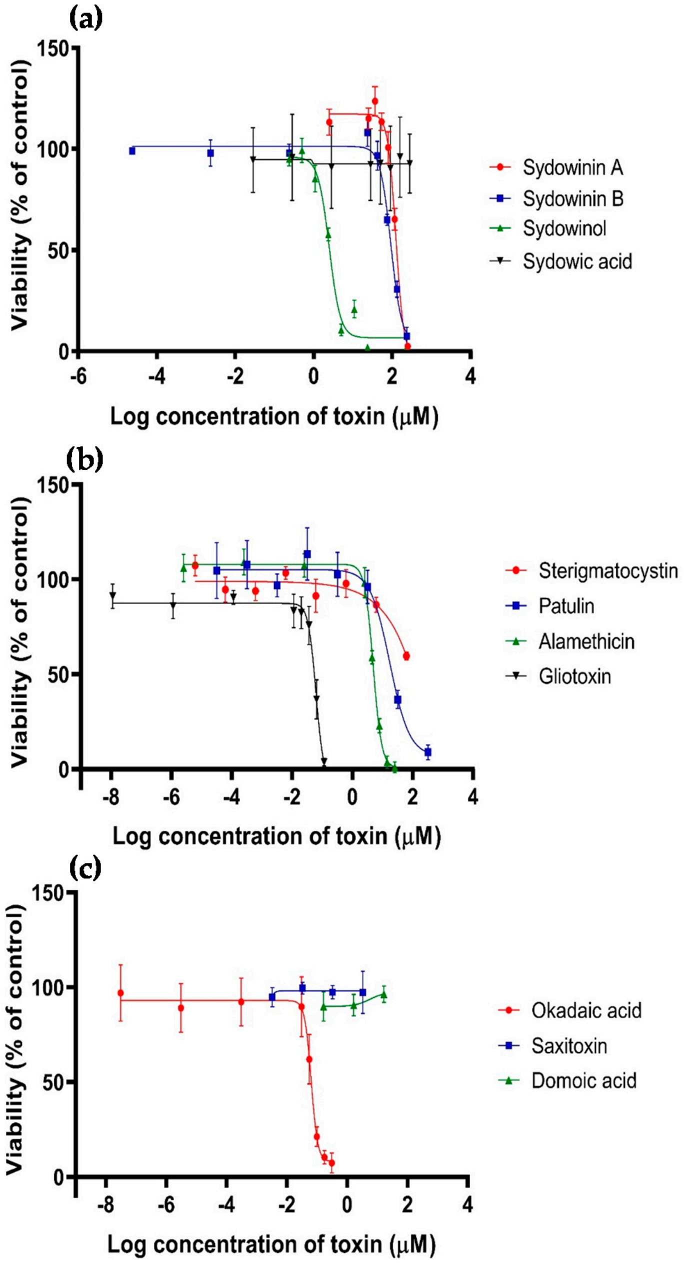

Figure S1. Dose–response curves of (**a**) major *A. sydowii* metabolites, (**b**) dust storm/shellfish-associated mycotoxins, and (**c**) algal toxins on human intestinal HT-29 cells. Data are mean ± SD of four replicates.

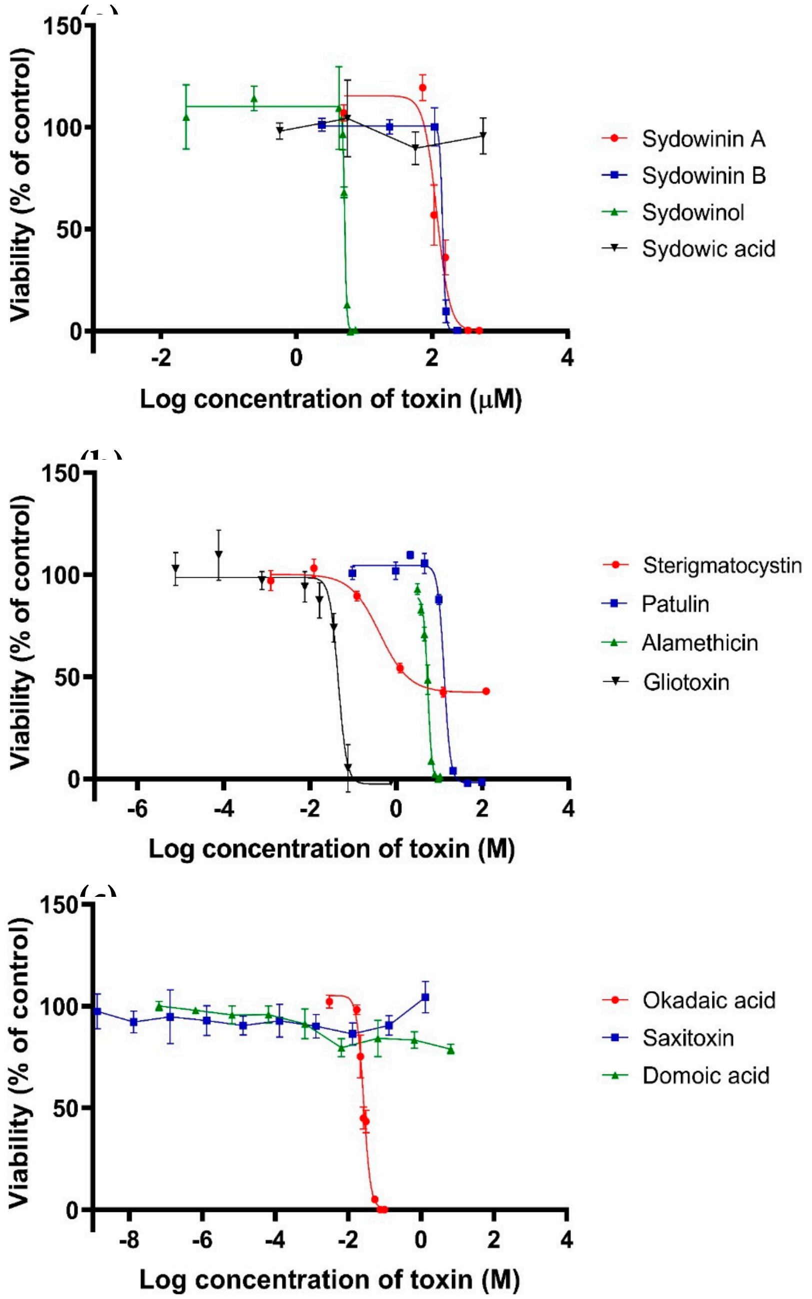

Figure S2. Dose–response curves of (**a**) major *A. sydowii* metabolites, (**b**) dust storm/shellfish-associated mycotoxins, and (**c**) algal toxins on human neuroblastoma SH-SY5Y cells. Data are mean ± SD of four replicates.

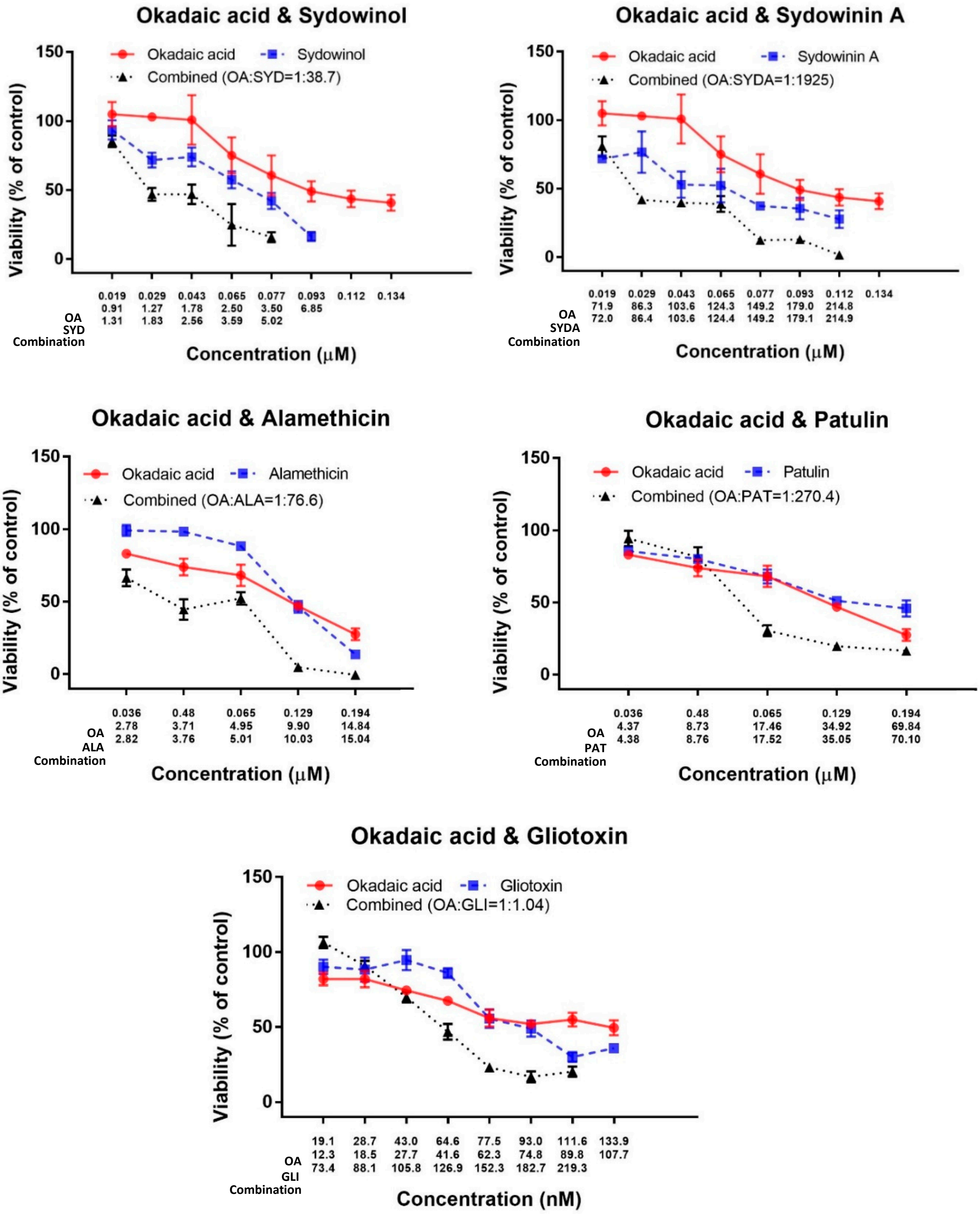

Figure S3. Okadaic acid (OA), sydowinol (SYD), sydowsyinin A (SYDA), alamethicin (ALA), patulin (PAT), and gliotoxin (GLI) and their binary mixture dose–responses for cytotoxicity against human intestinal HT-29 cell line. Concentrations in combinations were expressed as the sum of the concentrations of two toxins. Data are mean ± SD of four replicates.

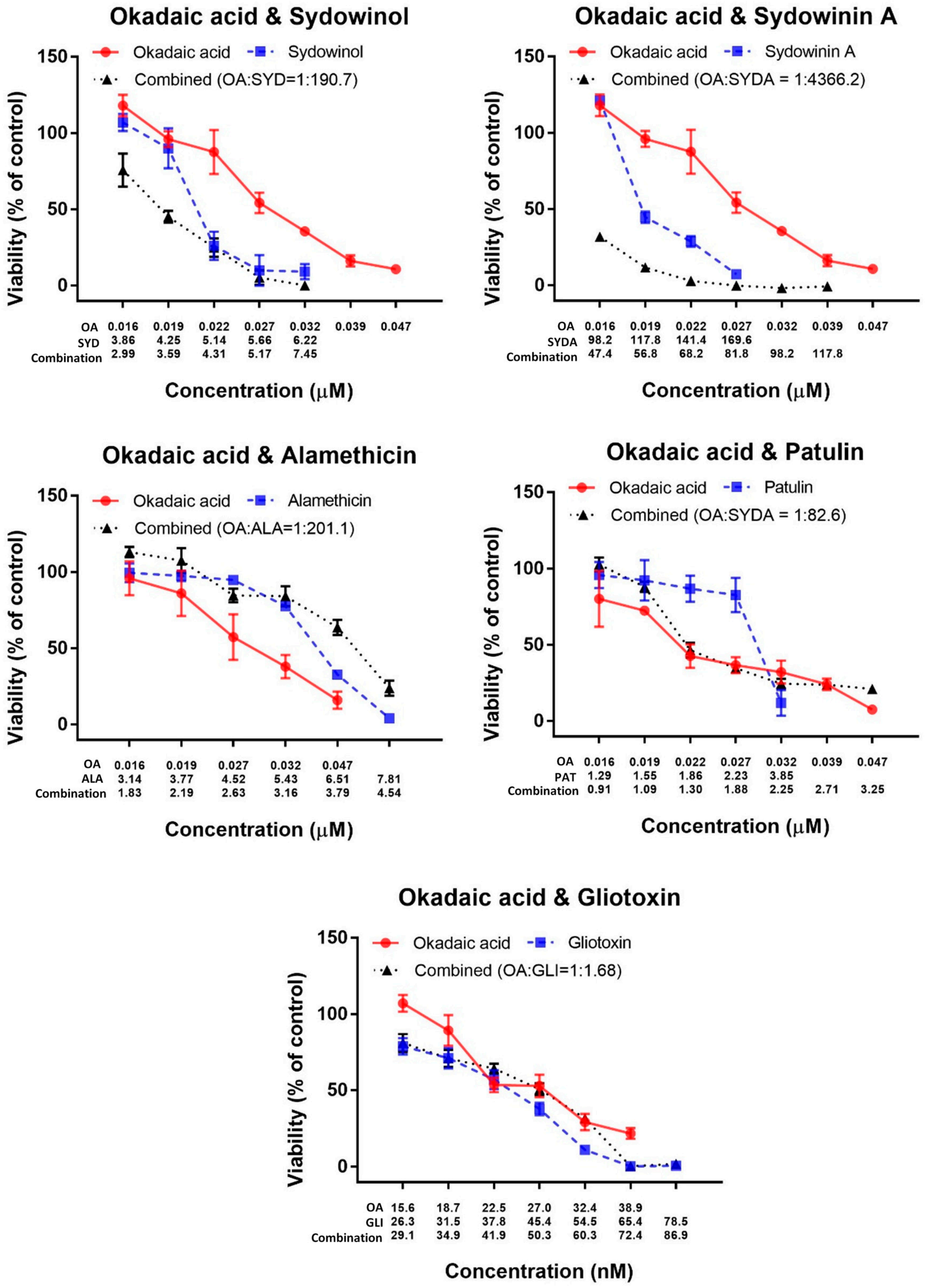

Figure S4. Okadaic acid (OA), sydowinol (SYD), sydowinin A (SYDA), alamethicin (ALA), patulin (PAT), and gliotoxin (GLI) and their binary mixture dose–responses for cytotoxicity against human neuroblastoma SH-SY5Y cell line. Concentrations in combinations were expressed as the sum of the concentrations of two toxins. Data are mean ± SD of four replicates.
